# Supplementary material for: The influence of green human resource management on university sustainability in higher education: the role of mediating environmental performance and green commitment
Source: PeerJ. 2024 Sep 18;12:e17966. doi: 10.7717/peerj.17966 (PMC11416077; doi:10.7717/peerj.17966)
Supplement: Supplemental Information 1 [file peerj-12-17966-s001.docx]

**Questionnaire**

| **Green Training, Involvement and Development (GTID)** | | |
| --- | --- | --- |
| 1. | | Providing environmental training to the organizational members to increase environmental awareness |
| 2. | | Take into account the needs of environmental issues when training requirement analyzed. |
| 3. | | Following Induction programs that emphasize environmental issues concerns. |
| 4. | | All training materials are available online for employee to reduce paper cost. |
| 5. | | Environmental training is a priority when compared to other types of company training. |
| **Green Recruitment and Selection (GRS)** | | |
| 6. | | Job description specification includes environmental concerns. |
| 7. | | Environmental performance of the company attracts highly qualified employees. |
| 8. | | Selecting applicants who are sufficiently aware of greening to fill job vacancies. |
| 9. | | Recruitment messages include environmental behavior/commitment criteria. |
| 10. | | Jobs positions designed to focus exclusively on environmental management aspects of the organizations. |
| **Green motivation(GM)** | | |
| 11. | Employees are encouraged by the top management to engage in environmental improvement. | |
| 12. | I feel happy when I am working intensely on environmental tasks. | |
| 13. | Incentives or reward are given to encourage environmental behaviour. | |
| 14. | Engagement workshops or forums are provided for staff to improve their environmental behaviour. | |
| 15. | Sufficient training is provided to employeesso that they can participate in environmental improvement efforts. | |
| **Green Performance Management and Appraisal (GPMA)** | | |
| 16. | Employees know their specific green targets, goals and responsibilities. | |
| 17. | Environmental behavior/targets and Contributions to environmental management are assessed and include in Performance indicators/appraisal and recorded. | |
| 18. | Roles of manages in achieving green outcomes included in appraisals. | |
| 19. | Corporate Incorporates environmental management objectives and targets with the performance evaluation system of the organization. | |
| 20. | Providing regular feedback to the employees or teams to achieve environmental goals or improve their environmental performance. | |
| **Environmental Orientation(EO)** | | |
| 21. | At our university, we make a concerted effort to let every employee understand the importance of environmental preservation. | |
| 22. | Our university has a clear policy statement urging environmental awareness in every area of operation. | |
| 23. | Environmental preservation is highly valued by our university members. | |
| 24. | Preserving the environment is a central corporate value in our university. | |
| 25. | Providing opportunities to the employee to involve and participate in green suggestion schemes and Joint consultations for environmental issues problem solving | |
| **Top Management Support (TMP)** | | |
| 26. | Top management at our university treats Green Human Resource Management as an important issue. | |
| 27 | Top management at our university allocates adequate resources to Green Human Resource Management efforts. | |
| 28 | Top management at our university allows employees to spend time on Green Human Resource Management efforts. | |
| 29 | Top management at our university follows up suggestions for improvement on Green Human Resource Management. | |
| 30 | Our university frequently use Green Human Resource Management teamwork to solve Green Human Resource Management problems. | |
| **Environmental Performance (EP)** | | |
| 31 | This team member suggests new ways to accomplish environmental goals. | |
| 32 | This team member propose new green ideas to improve environmental performance. | |
| 33 | This team member promote and champion new green ideas to others. | |
| 34 | This team member develops adequate plans for the application of new green ideas. | |
| 35 | This team member would rethink new green ideas. | |
| **Green Commitment (GM)** | | |
| 36. | Top Management Commitment is committed to protecting nature. | |
| 37. | Top Management Commitment actively support the environmental friendly initiatives. | |
| 38. | Top Management Commitment clarifies information and values of environmental management. | |
| 39. | Top Management Commitment established penalties for noncompliance in the environmental management. | |
| 40. | Top Management Commitment involved /participate in environmental projects, | |
| **University Sustainability (US)** | | |
| 41. | Improved overall stakeholder welfare. | |
| 42. | Improvement in community health and safety. | |
| 43. | Reduction in environmental impacts and risks to the general public. | |
| 44. | Improved occupational health and safety of employees. | |
| 45. | University vision/mission statements include environmental concern. | |
