# Supplementary material for: The influence of green human resource management on university sustainability in higher education: the role of mediating environmental performance and green commitment
Source: PeerJ. 2024 Sep 18;12:e17966. doi: 10.7717/peerj.17966 (PMC11416077; doi:10.7717/peerj.17966)
Supplement: Supplemental Information 2 [file peerj-12-17966-s002.docx]

الاستبيان

| التدريب الأخضر والمشاركة والتطوير | |
| --- | --- |
| **توفير التدريب البيئي لأعضاء المنظمة لزيادة الوعي البيئي.** | **1** |
| **مراعاة احتياجات القضايا البيئية عند تحليل متطلبات التدريب.** | **2** |
| **اتباع البرامج التعريفية التي تركز على القضايا البيئية.** | **3** |
| **جميع المواد التدريبية متاحة للموظفين عبر الإنترنت لتقليل تكلفة الورق.** | **4** |
| **يعد التدريب البيئي أولوية عند مقارنته بأنواع التدريب الأخرى في الشركة.** | **5** |
| التوظيف والاختيار الأخضر | |
| **تتضمن مواصفات الوصف الوظيفي الاهتمامات البيئية.** | **6** |
| **الأداء البيئي للشركة يجذب الموظفين المؤهلين تأهيلا عاليا.** | **7** |
| **اختيار المتقدمين الذين لديهم المعرفة الكافية بالتخضير لشغل الوظائف الشاغرة.** | **8** |
| **تتضمن رسائل التوظيف معايير السلوك/الالتزام البيئي.** | **9** |
| **وظائف مصممة للتركيز حصريًا على جوانب الإدارة البيئية للمنظمات.** | **10** |
| الدافع الأخضر | |
| **يتم تشجيع الموظفين من قبل الإدارة العليا للمشاركة في تحسين البيئة.** | **11** |
| **أشعر بالسعادة عندما أعمل بشكل مكثف على المهام البيئية.** | **12** |
| **يتم تقديم الحوافز أو المكافآت لتشجيع السلوك البيئي.** | **13** |
| **يتم توفير ورش عمل أو منتديات للمشاركة للموظفين لتحسين سلوكهم البيئي.** | **14** |
| **يتم توفير التدريب الكافي للموظفين حتى يتمكنوا من المشاركة في جهود تحسين البيئة.** | **15** |
| إدارة وتقييم الأداء الأخضر | |
| **يعرف الموظفون أهدافهم وأهدافهم ومسؤولياتهم الخضراء المحددة.** | **16** |
| **يتم تقييم السلوك البيئي/الأهداف والمساهمات في الإدارة البيئية وإدراجها في مؤشرات/تقييم الأداء وتسجيلها.** | **17** |
| **أدوار الإدارة في تحقيق النتائج الخضراء المدرجة في التقييمات.** | **18** |
| **تقوم الشركة بدمج أهداف وغايات الإدارة البيئية مع نظام تقييم أداء المنظمة.** | **19** |
| **تقديم تعليقات منتظمة للموظفين أو فرق العمل لتحقيق الأهداف البيئية أو تحسين أدائهم البيئي.** | **20** |
| التوجه البيئي | |
| **في جامعتنا، نبذل جهودًا متضافرة للسماح لكل موظف بفهم أهمية الحفاظ على البيئة.** | **21** |
| **لدى جامعتنا بيان سياسة واضح يحث على الوعي البيئي في كل مجال من مجالات العمل.** | **22** |
| **يحظى الحفاظ على البيئة بتقدير كبير من قبل أعضاء جامعتنا.** | **23** |
| **يعد الحفاظ على البيئة قيمة مؤسسية مركزية في جامعتنا.** | **24** |
| **توفير الفرص للموظف للانخراط والمشاركة في خطط الاقتراحات الخضراء والمشاورات المشتركة لحل المشكلات المتعلقة بالقضايا البيئية.** | **25** |
| دعم الإدارة العليا | |
| **تتعامل الإدارة العليا في جامعتنا مع إدارة الموارد البشرية الخضراء باعتبارها قضية مهمة.** | **26** |
| **تخصص الإدارة العليا في جامعتنا الموارد الكافية لجهود إدارة الموارد البشرية الخضراء.** | **27** |
| **تسمح الإدارة العليا في جامعتنا للموظفين بقضاء بعض الوقت في جهود إدارة الموارد البشرية الخضراء.** | **28** |
| **تتابع الإدارة العليا في جامعتنا اقتراحات تحسين إدارة الموارد البشرية الخضراء.** | **29** |
| **تستخدم جامعتنا في كثير من الأحيان العمل الجماعي لإدارة الموارد البشرية الخضراء لحل مشاكل إدارة الموارد البشرية الخضراء.** | **30** |
| الأداء البيئي | |
| **يقترح عضو الفريق هذا طرقًا جديدة لتحقيق الأهداف البيئية.** | **31** |
| **يقترح عضو الفريق هذا أفكارًا خضراء جديدة لتحسين الأداء البيئي.** | **32** |
| **يقوم عضو الفريق هذا بتعزيز ودعم الأفكار الخضراء الجديدة للآخرين.** | **33** |
| **يقوم عضو الفريق هذا بتطوير خطط مناسبة لتطبيق الأفكار الخضراء الجديدة.** | **34** |
| **سيعيد عضو الفريق هذا التفكير في الأفكار الخضراء الجديدة.** | **35** |
| الالتزام الأخضر | |
| **يلتزم التزام الإدارة العليا بحماية الطبيعة.** | **36** |
| **التزام الإدارة العليا يدعم بنشاط المبادرات الصديقة للبيئة.** | **37** |
| **يوضح التزام الإدارة العليا معلومات وقيم الإدارة البيئية.** | **38** |
| **التزام الإدارة العليا وضع عقوبات لعدم الامتثال في الإدارة البيئية.** | **39** |
| **التزام الإدارة العليا بالمشاركة/المشاركة في المشاريع البيئية.** | **40** |
| استدامة الجامعة | |
| **تحسين الرفاهية العامة لأصحاب المصلحة.** | **41** |
| **تحسين صحة المجتمع وسلامته.** | **42** |
| **الحد من الآثار البيئية والمخاطر التي يتعرض لها عامة الناس.** | **43** |
| **تحسين الصحة والسلامة المهنية للموظفين.** | **44** |
| **تتضمن بيانات رؤية/رسالة الجامعة الاهتمامات البيئية.** | **45** |
